# Altered Synchronizations among Neural Networks in Geriatric Depression

**DOI:** 10.1155/2015/343720

**Published:** 2015-06-09

**Authors:** Lihong Wang, Ying-Hui Chou, Guy G. Potter, David C. Steffens

**Affiliations:** ^1^Department of Psychiatry, University of Connecticut Health Center, 263 Farmington, CT 06119, USA; ^2^Department of Psychiatry and Behavioral Sciences, Duke University, Durham, NC, USA; ^3^Brain Imaging and Analysis Center, Duke University, Durham, NC, USA

## Abstract

Although major depression has been considered as a manifestation of discoordinated activity between affective and cognitive neural networks, only a few studies have examined the relationships among neural networks directly. Because of the known disconnection theory, geriatric depression could be a useful model in studying the interactions among different networks. In the present study, using independent component analysis to identify intrinsically connected neural networks, we investigated the alterations in synchronizations among neural networks in geriatric depression to better understand the underlying neural mechanisms. Resting-state fMRI data was collected from thirty-two patients with geriatric depression and thirty-two age-matched never-depressed controls. We compared the resting-state activities between the two groups in the default-mode, central executive, attention, salience, and affective networks as well as correlations among these networks. The depression group showed stronger activity than the controls in an affective network, specifically within the orbitofrontal region. However, unlike the never-depressed controls, geriatric depression group lacked synchronized/antisynchronized activity between the affective network and the other networks. Those depressed patients with lower executive function has greater synchronization between the salience network with the executive and affective networks. Our results demonstrate the effectiveness of the between-network analyses in examining neural models for geriatric depression.

## 1. Introduction

It has long been postulated that major depression may be a consequence of failed coordination between the central executive system and affective processing system [1]. While a great number of studies [2, 3] have identified abnormal activation in the regions subserving executive function (e.g., dorsolateral prefrontal cortex (dlPFC) and dorsal anterior cingulate (dACC)) and affective processing (e.g., ventromedial prefrontal cortex (vmPFC), orbitofrontal cortex (OFC), and amygdala), only a few studies have examined coordination between the executive system and affective processing system directly at a network level in major depression.

In addition to the executive and affective processing systems, the abnormalities in the default-mode network (DMN, primarily including the anterior and posterior cingulate, and bilateral lateral parietal cortex areas) and salience network (including the dorsal anterior cingulate and insula cortices) in major depression have also been identified [4]. A number of studies have reported an increased activity of the DMN in major depression during resting state [5, 6] and persistent activity of the DMN during tasks [4, 7]. Northoff and Sibille [8] have suggested hyperactivity of the DMN as one of the endophenotypes of major depression, which could predispose individuals with this endophenotype to depression, whereas Marchetti and colleagues [9] hypothesized that the increased DMN activity could be a depressive scar resulting from a dysfunctional switch between internally and externally oriented attention. Meanwhile, there are also reports about decreased function in the executive system [2, 3, 10] that some authors refer to as task-positive deficiency [9, 11]. Interestingly, Hamilton and colleagues [4] have reported a task-negative (i.e., DMN) dominance over task-positive network dominance during resting state in major depression using an index to quantify the number of time periods when the DMN signal is stronger than the signal from the task-positive network. They also proposed that the right anterior insula might be a driver subserving the switch between internal and external attentions. They found that while in healthy controls the anterior insula activity was increased when task-positive activity was at the peak, in patients with major depression the anterior insula activity was increased when the DMN activity was at the peak. The anterior insula as a key node of the salience network (SN) has recently gained lots of attention in the neuroimaging research field and has been found also involved in major depression by many other authors [12–14]. van Tol et al. [13] reported decreased functional connectivity of the salience network with the medial prefrontal cortex, ventrolateral prefrontal cortex, and ventral striatum. Manoliu and colleagues [12] recently also found decreased connectivity between the insula and dorsal anterior cingulate (dACC) within the salience network, which was associated with the severity of symptoms and aberrant DMN/CEN interactions as well. Furthermore, Yuen and colleagues [14] found the decreased right anterior insular-dACC connectivity and increased insular-dorsolateral prefrontal cortex (dlPFC) connectivity in older major depression patients who had apathy symptoms. Therefore, it is necessary to clarify the relationships among the affective, executive, DMN, attention, and salient systems in major depression.

Major depression in individuals who had the first depression episode at their older ages (typically older than 50 years) is often referred as geriatric depression. Different from major depression in younger adults, geriatric depression has frequently been found in those with cerebrovascular disorders [15, 16], such as white matter hyperintensities, which are associated with disconnections/low blood supplies in white matters and gray matters. Because of the disconnection pathology, geriatric depression could serve as an interesting model in studying the alteration of the interactions among different neural networks. However, so far, there is only one study in geriatric depression which has investigated the interaction between regions from different neural networks. That study was focused only on the differences of the insular connectivity between those who had high (*n* = 7) versus low (*n* = 9) apathy symptoms, which needs further confirmation in a larger sample. Therefore, more studies in geriatric depression are very necessary.

In recent years, with the development of various analyzing methods on task-related and task-free functional magnetic resonance imaging (fMRI) data, analyzing fMRI data at a neural network level becomes a reality. One of the widely used techniques to identify neural networks is the independent component analysis (ICA). Unlike the seed-based functional connectivity analysis which is dependent on the location and size of a seed, the ICA approach is data driven. It identifies independent components (ICs) based on the spatial and temporal distribution patterns [17]. Since the regions within an IC are temporally synchronized and are commonly activated during a certain cognitive processing simultaneously, these regions within an IC are often considered to be within the same neural network. A number of studies [18–20] have identified intrinsically connected neural networks by comparing ICs with task-activated brain regions through meta-analysis. With the identified neural networks, we can further investigate the properties of the neural networks and the relationships among different neural networks.

The majority of previous studies in the literature have examined the association of regional activity or connectivity between two regions with depression severity [4, 6, 21, 22]. Although there are some reports on interactions among regions from different neural networks [23], few studies have examined the interactions among different neural networks by evaluating the synchronization of an entire network. The advantage of evaluating the synchronization of an entire network over the region-to-region synchronization analysis is that the former would allow us to compute/understand a neural model for a mental disorder more easily. To examine the interactions between neural networks in geriatric depression, we conducted an ICA and identified the default-mode, executive, attention, affective, and salience networks by comparing each component with the template of Laird and colleagues [19] using the goodness of fit analysis. Then we computed the significant differences between the depressed and healthy control groups in the correlations among these networks. Regression analyses between the network synchronizations with depression severity were also conducted. Since geriatric depression typically has executive dysfunction, we conducted the study in geriatric depression to examine the influence of network interactions on depressive symptoms and executive function. We hypothesized that the correlation/coordination between or among networks rather than in a single network has a strong association to depressive symptoms and executive dysfunction.

## 2. Materials and Methods

### 2.1. Participants

Thirty-two individuals who had been diagnosed with major depressive disorder (19 females, mean ± SD age: 68 ± 6.5 years) and thirty-two healthy never-depressed volunteers (18 females, mean ± SD age: 72 ± 8.2 years) participated in this study. Participants were recruited from the neurocognitive outcomes of depression in the elderly study (NCODE). All depressed patients met DSM-IV criteria for major depression. They were either in a remitted state (*n* = 21) or in an actively depressed state (*n* = 11) with the Montgomery-Åsberg Depression Rating Scale (MADRS) mean ± SD score of 2.1 ± 1.8 for the remitted and 17.4 ± 9.2 for the actively depressed patients. The exclusion criteria for depressed subjects included (1) another major psychiatric illness, including bipolar disorder, schizophrenia, or dementia; (2) alcohol or drug abuse or dependence; (3) neurological illness, including dementia, stroke, and epilepsy; (4) medical illness, medication use, or disability that would prevent the participant from completing neuropsychological testing; and (5) contraindications to MRI. All never-depressed subjects were cognitively intact and had no history or clinical evidence of dementia, and they all scored 28 or more on the minimental state examination. Among the 32 depressed participants, 9 were receiving antidepressant monotherapy (4 on selective serotonin reuptake inhibitors (SSRIs), 2 on serotonin antagonist and reuptake inhibitors (SARIs), 1 on serotonin-norepinephrine reuptake inhibitors (SNRIs), and 2 on an tricyclic), 9 were receiving combination treatment (4 on two SSRIs, 2 on SSRI combined with either SARI or norepinephrine-dopamine reuptake inhibitors (NDRIs), 2 on SARI and NDRI, and 1 on SNRI and NDRI), and 14 were not on medication (Table 1).

Prior to the fMRI, all subjects completed the Stroop Color and Word Test to examine the executive function. The study received approval by Duke School of Medicine Institutional Review Board. All subjects gave verbal and written consent after being explained the purpose and procedures to be used in the study.

### 2.2. Neuroimaging Acquisition

All participants were scanned using a research-dedicated 3.0 T GE EXCITE HD scanner (GE Medical Systems, Milwaukee, Wisconsin). First, high-resolution T1-weighted structural images in coronel view were acquired with slice thickness of 1 mm without a gap (matrix = 256 × 256 × 216). We then obtained 5-minute resting fMRI scans for each participant. Participants were instructed to rest without moving, keep their eyes open, and focus on a fixation cross-presented in the center of the screen inside the scanner. Inward spiral sequence functional images in the axial view were acquired using the following parameters: TR = 2000 ms, TE = 31 ms, FOV = 24 cm, flip angle = 90°, and matrix = 64 × 64 × 34.

### 2.3. Data Analyses

Data were preprocessed using the Duke BIAC resting state pipeline based on the tools from the FSL analysis package (FMRIB Software Library, http://fsl.fmrib.ox.ac.uk/fsl/fslwiki/, Version 5.98) and locally developed MATLAB code (MathWorks, Natick, MA), including slice-timing alignment, motion correction, coregistration, nonbrain voxel extraction, and normalization. We also regressed out six-parameter rigid body head motion, the signal averaged over the white matter, and signal averaged over the cerebrospinal fluid regions [24]. Frequencies less than 0.08 Hz were retained [25]. The group independent component analysis (ICA) was conducted using melodic and dual regression program following the instructions on FslWiki (http://fsl.fmrib.ox.ac.uk/fsl/fslwiki/DualRegression). Briefly, we first concatenated all subjects' data including both the patient and control groups and calculated the group-averaged independent components (IC) by limited the ICs to twenty components to match the study of Laird and colleagues [19]. Next, we identified the default-mode network (DMN), central executive network (CEN), central attentional network (CAN), salience network (SN), and affective network (AN) by using the goodness of fit test (GOF) [5] to best match the networks provided by Laird and colleagues [19]. In the case that the second largest GOF value was close to the first largest GOF value, we kept the component as a component of interest as well. Next, we regressed spatial ICs into each subject's 4D data to generate both subject-specific component time courses and subject-specific spatial maps as outputs [26]. Specifically, as described in the FSL webpage (http://fsl.fmrib.ox.ac.uk/fsl/fslwiki/DualRegression), “for each subject, the group-average set of spatial maps is regressed (as spatial regressors in a multiple regression) into the subject's 4D space-time dataset. This results in a set of subject-specific timeseries [sic], one per group-level spatial map. Next, those timeseries [sic] are regressed (as temporal regressors, again in a multiple regression) into the same 4D dataset, resulting in a set of subject-specific spatial maps, one per group-level spatial map.” The z-score for every voxel was estimated by normalizing each voxel's intensity with respect to intensity of all the voxels in each individual IC. The IC maps were then compared between groups on a voxelwise basis for statistical tests using FSL's randomize permutation-testing tool. Finally, pairwise Pearson's correlation coefficient analyses were conducted to compute the interaction between any two networks as listed above.

### 2.4. Statistical Analyses

To identify group difference between the depression patients and controls in each component identified as DMN, CEN, CAN, SN, and AN and the inter-IC correlations between any two networks, voxelwise two-sample* t*-tests were computed. Two-sample* t*-tests were conducted to test group differences in inter-IC correlations. Age was used as a regressor to control the aging effect. To examine the interactions between network activity strength and clinical status, we also conducted regression analyses using MADRS score (depression severity) and executive function as measured by the color-word interference condition of the Stroop task. The measures of the Stroop task were converted into standardized score based on age, gender, and race. Significant level was determined using threshold of *Z* > 2.3, *P* < 0.05 with cluster correction. For the interactions between networks, the significance was determined using *P* < 0.05 based on the Monte-Carlo simulation. Specifically, similar to the network-based statistics [27], our multiple comparisons were corrected based on nonrandom data distribution patterns. The first step was to identify a set of correlations that exhibited a *P* value less than 0.05. Second, among the set of correlations, we determined whether a cluster of correlations was significant based on the size of the cluster. The size of the cluster was determined by 10,000 Monte-Carlo simulations.

## 3. Results

### 3.1. Clinical Profile of the Participants

We summarize the demographic details, clinical profile, and performance of participants on the Stroop task in Table 1. Given a nonsignificant trend for age difference between the two groups, with the control group relatively older than the depression group, we included age as a covariate in subsequent group comparison analyses. For executive function, the depression group showed a relatively lower score than the control group in performing the Stroop task; however, there was not a significant difference between the two groups (Table 1).

### 3.2. Differences in Resting-State Activity within and between Neural Networks between Depression and Control Groups

**Figure 1:**
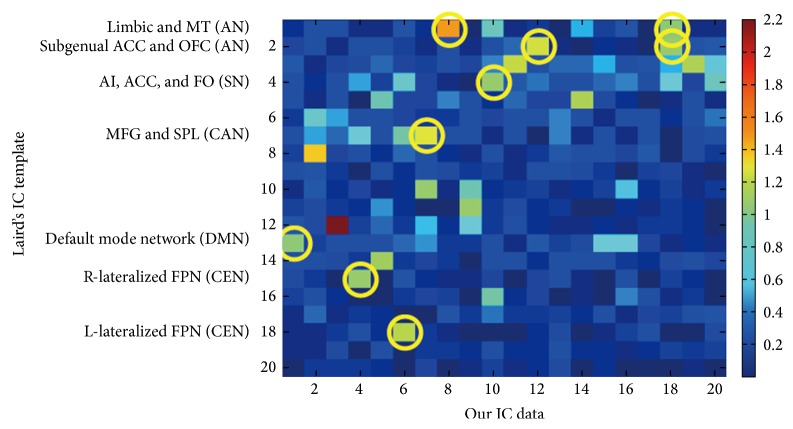
The ICA components which correspond to different neural networks according to the goodness-of-fit analysis using the templates of Laird et al. AN = affective network; CAN = central attentional network; CEN = central executive network; DMN = default-mode network; SN = salience network.

**Figure 2:**
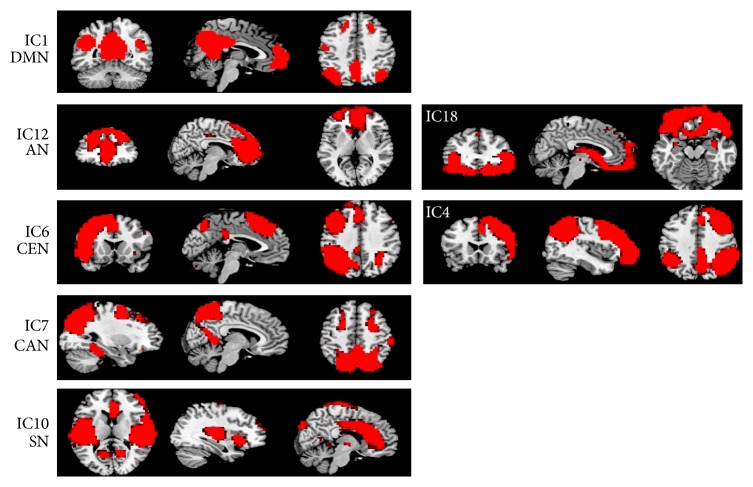
The locations of each IC component which correspond to different neural networks according to the goodness-of-fit analysis using the templates of Laird et al. The IC components were computed using dual regression analysis by combining the data from both the depression and never-depressed control group (*Z* > 2.3, *P* < 0.05 with cluster correction).

Our aim was to examine whether we can identify altered interactions among networks that are related to depressive symptoms and cognitive dysfunctions in geriatric depression. To achieve this goal, using the results from Laird and colleagues [19] as templates, first we identified the components that were best matched to the default-mode network (DMN, IC1, corresponding to Laird et al.'s IC13), central executive network (CEN, IC4, and IC6 corresponding to Laird et al.'s IC15 and IC18, resp.), central attentional network (CAN, IC7 corresponding to Larid et al.'s IC7), salience network (SN, IC10, corresponding to Laird et al.'s IC4), and affective work (AN, IC12, and IC18, corresponding to Laid et al.'s IC2; the IC18 was also matched to Laird et al.'s IC1).  Figure 1 shows the matched components between the ICs in our study with Larid et al.'s. The detailed coverage for each component is listed in Table 2 and Figure 2. More detailed coverage of each component is shown in axial views in supplementary sFigure 1 in Supplementary Material available online at http://dx.doi.org/10.1155/2015/343720.

**Figure 3:**
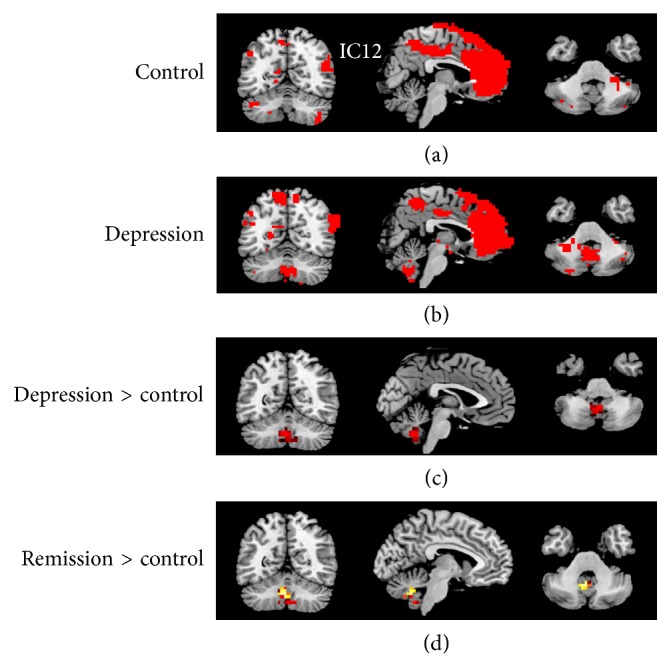
(a) Component 12 (IC12), one of the affective networks, in the control group; (b) IC12 in the depression group. To show the voxels in the cerebellum, (a) and (b) were based on threshold of *Z* > 2.3, *P* < 0.001 without cluster correction. (c) Regions within IC12 which showed significantly increased activity in the depression group (all patients) related to the control group; (d) regions within IC12 which showed significantly increased activity in subjects remitted from depression (part of patients in the depression group) related to the controls. (c) and (d) were based on threshold of *Z* > 2.3, *P* < 0.05 with cluster correction.

When comparing each individual network between patients and controls using two-sample* t*-tests, we found significantly increased IC12 (one of the affective networks, ANs) activity specifically in the cerebellar vermis in the depression group relative to the control group. In fact, the cerebellar vermis was not represented in the IC12 when we use the threshold of *Z* > 2.3, *P* < 0.05 with cluster correction. However, the IC12 of the depressed group did have a cluster in the cerebellar vermis when using the threshold of *Z* > 2.3, *P* < 0.001 without cluster correction (Figure 3).

**Figure 4:**
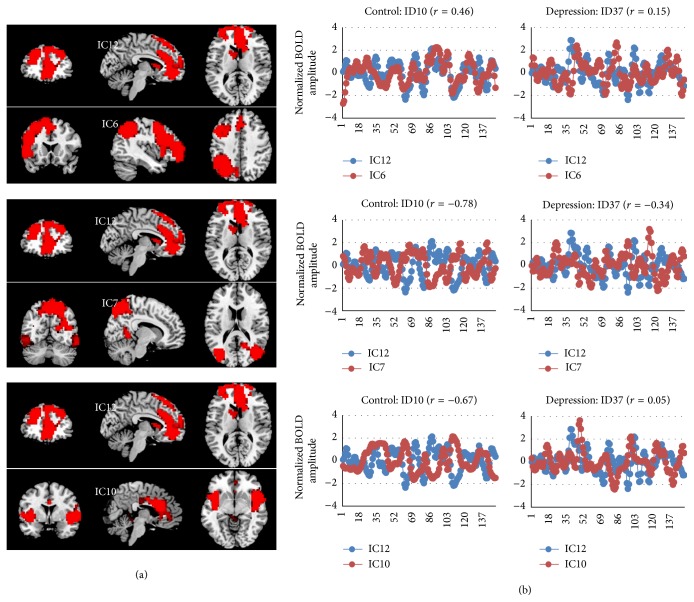
(a) Neural network pairs which revealed significant correlations in the control group. (b) The plots of time course of each network in a control subject (ID10) and a depression subject (ID37) to illustrate the interaction effect between each paired neural networks. The significance was tested using Monte-Carlo simulation.

When comparing correlations among networks between the two groups (pairwise correlations), it was the IC12 that showed a significant group difference in the synchronizations between this network with several other networks (Table 3). Specifically, we found a positive correlation in the IC12 with IC6 (one of the CENs) in the control group; however, the correlation was significantly reduced (no significant correlation existed) in the depression group. We also found a significantly increased correlation (less negative) in the depression group relative to the control group in the IC12 with IC7 (CAN, mainly in the precuneus region) and the IC12 with IC10 (the salience network, SN) (Figure 4).

### 3.3. Correlation with Depression Severity and Executive Function

**Figure 5:**
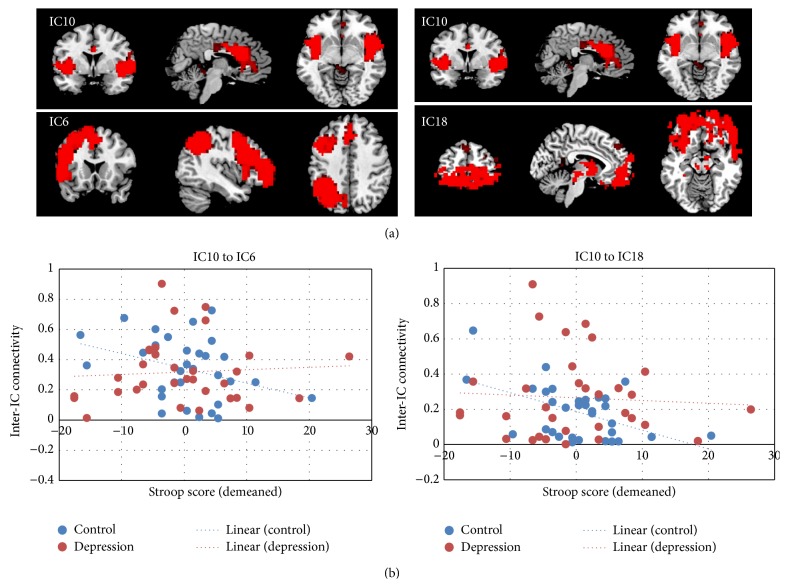
(a) Neural network pairs that their correlation or anticorrelation was correlated with the Stroop task performance in depression patients. (b) The correlation plots in the patient group (red) and control group (blue).

We did not find any significant correlation of the severity of depressive symptoms with any of the networks or interactions between any two networks. However, as shown in Figure 5, we found a negative correlation between the Stroop task performance and the interactions between the IC10 (SN) and IC6 (one of the CENs) and between IC10 (SN) and IC18 (one of the AN) in the depression group but not in the control group. In other words, in the depression group, those who had higher synchronization between the salience network and the central executive network and affective networks were the ones who had poor task performance on the Stroop task.

### 3.4. Depression-State Related Alteration

**Figure 6:**
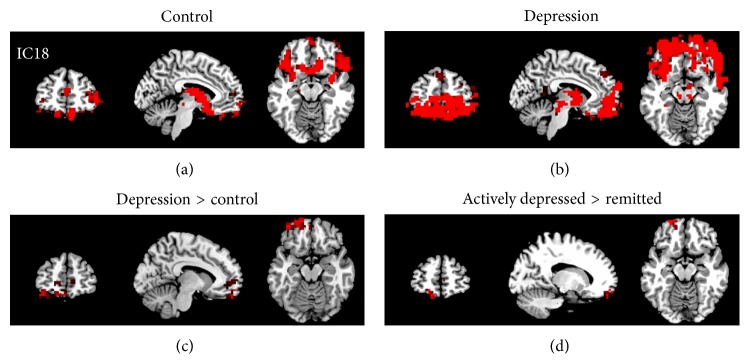
Upper: the IC18 in the control group (a) and depression group. (b) Lower: the regions within IC18 that revealed significantly increased activity in the depressed patients than controls (c) and in the actively depressed patients than the remitted patients (d) (*Z* > 2.3, *P* < 0.05 with cluster correction).

Given the fact that we had a fair number of patients in a remitted state, we suspected that the reason that we did not find a significant correlation between neural activity and depression severity might be because their relationship is nonlinear; that is, it might be a depression-state dependent rather than a linear relationship. Therefore, we subsequently examined the differences in neural networks and interactions of networks between the remitted versus the actively depressed groups and between the remitted versus the control groups. As shown in Figures 3(c) and 3(d), the increased cerebellar vermis activity shown in the pooled depression group relative to controls was mainly driven by the remitted group in comparison with controls. The increased cerebellar vermis activity was not found in the actively depressed group in comparison with the control group. Instead, we found significantly increased resting activity of IC18 (another AN) in the left orbitofrontal cortex and ventromedial prefrontal cortex in the actively depressed group compared with both the control group and the remitted patient group (Figure 6). Therefore, we believe the increased orbitofrontal cortex of AN should be a depression-state effect.

We also examined the group differences in network synchronizations between the actively depressed versus control, actively depressed versus remitted, and remitted versus control groups. The analyses confirmed the network interaction results in the combined patient sample, in that the significant positive correlation between IC10 (SN) and IC6 (CEN) and the negative correlation between IC10 (SN) and IC7 (CAN) in the control group were significantly less positive or less negative in the actively depressed group. There were no significant group differences between the actively depressed and remitted groups or between the remitted and control groups among the network interactions.

## 4. Discussion

We investigated the interactions among different intrinsic connectivity networks in patients with both acute and remitted geriatric depression and found that depression patients had significant alterations in the synchronizations/antisynchronizations between the affective network with other networks including the central executive network, attentional network, and the salience network. In addition, we found depressive-state specific increase in the orbitofrontal area of the affective network. Although these changes were not correlated with depression severity, the significant differences confirmed in the acutely depressed group indicate an importance of the interactions between networks as the neuropathology of major depression.

It is interesting that the depression group mainly had altered correlations between the component of the affective network (including the orbitofrontal, subgenual cingulate, and the dorsomedial prefrontal cortex) with other neural networks. This component best matched component 2 (subgenual cingulate and orbitofrontal cortex) of Laird and colleagues' study and the authors indicated its role in “olfaction, gustation, and emotion” [19]. Previous studies in the literature using task-related fMRI have frequently found activation in the orbitofrontal and the dorsomedial prefrontal cortex during emotion related tasks, particularly in tasks related to emotion expectation and emotional learning [28–30]. It is hypothesized that medial prefrontal cortex (mPFC) may use the inputs from the orbitofrontal cortex (OFC) as signals of internal states to select appropriate behaviors during automatic cognitive change paradigms [31]. In fact, in the model of Phillips and colleagues [31], the OFC, mPFC, and subgenual cingulate, together with the hippocampus and parahippocampus, could function as an automatic emotion regulation system. They also proposed the central executive system including dorsolateral prefrontal cortex, dorsomedial prefrontal cortex, and dorsal anterior cingulate as voluntary emotion regulation system. Phillips and colleagues pointed that the voluntary emotion regulation system function may be mediated by the OFC and subgenual cingulate [31]. Consistently, we found a positive correlation between the automatic emotion regulation system (IC12) and the voluntary emotion regulation system (IC6) in the healthy control group, the correlation did not exist in the depression group, suggesting that emotion regulation requires good coordination/synchronization between the automatic and voluntary systems. That is, the synchronization between the two systems was broken in depressed patients, which could result in depressive symptoms.

We also found a negative correlation between the IC12 with IC7 and IC10 in the healthy control group and the correlation become less negative in the depressed group. The IC7 located at the frontal eye area and the precuneus areas and best matched the IC7 of Laird's study which should be an attentional network, whereas IC10 matched the IC4 of Laird's study (the bilateral anterior insula/frontal opercula and ACC) which should be the salience network. The salience network recently has been hypothesized to play an important role in facilitating attentional transition between cognition and emotion/interoception [32, 33]. Negative correlations between the automatic emotional regulation system with the attentional system and the salient system suggest that when the automatic emotional regulation system was working, attention to affective stimuli and attentional transition from cognition to emotion/interoception would be suppressed, which could be a consequence of successful automatic emotional regulation. However, the network interactions disappeared in the depressed group. Together with the discoordination of the automatic emotion regulation system with the voluntary emotion regulation system, we speculate that the major deficits in our depression group were the discoordination between the affective (automatic emotion regulation) system and the other neural systems (central executive network, cognitive attention, and attention transition between cognition and emotion/interoception). Although we did not find a significantly linear correlation between the network interactions with depression severity, the subgroup analysis confirmed the results were more significant in the currently depressed group than the remitted group. Although our study sample was older adults, our findings are largely in consistent with Mayberg [1] and Philips's neuroscience model of depression and emotion.

Since we mainly found a discoordination between the affective networks with other networks, we speculate that the primary deficits in depression could be in the automatic emotion regulation system of the affective network which have resulted in the interaction deficits between this network and other networks. Indeed, we found increased activity in the left orbitofrontal cortex area (although not IC12 but IC18 instead) in depression, especially in the actively depressed group relative to both the remitted depression group and healthy control group (suggesting a depressive state-related alteration). As shown in the results, there are some spatial overlaps in the ventromedial prefrontal and orbitofrontal regions between the IC18 and IC12. Similar to IC12, the IC18 also matched the IC2 as well as IC1 of Lairds' study, but the IC18 also included the limbic and brainstem regions, all of which should also be part of the automatic emotion regulation network. The pathological deficit in the orbitofrontal cortex in depression has long been well documented [28]. Rajkowska et al. [34] found a decrease in cortical thickness of orbitofrontal cortex in depressed patients. In older adults, decreased volume of the orbitofrontal cortex has been reported in many studies [35–37]. Increased metabolism or regional cerebral blood flow (rCBF) of the orbitofrontal cortex has been shown in unmedicated depressive patients [38] although decreased orbitofrontal activation was found associated with anxiety symptoms [39]. Therefore, in consistent with Drevets and colleagues' theory [28], increased resting activity in the orbitofrontal area of the automatic emotion regulation network could be a core deficit in depression.

In this study, we also found a negative correlation between the Stroop task performance and the synchronization of the salience network (IC10) with the central executive network/voluntary emotion regulation network (IC6) as well as the synchronization of the salience network with the automatic emotion regulation network (IC18) in the depression group but not in the control group. In other words, those patients, who had poor performance in the Stroop task, had stronger synchronization between the salience network and the emotional regulation (both voluntary and automatic) networks. The results may implicate that, those whose salience network and emotional regulation networks are positively synchronized, may be more easily to reallocate their attention to emotional events, which then could distract them from ongoing cognitive tasks and result in poor performance in the executive tasks such as the Stroop task. Supportively, in the control group, the salience network was negatively correlated with the automatic emotion regulation network (IC12 though). However, it is difficult to explain why those who had poor performance during the Stroop task had a positive correlation between the salience network and the voluntary emotional regulation network. While it might be a compensatory effect, future studies to investigate the synchronization between the salience network and the voluntary emotional regulation (central executive) network during performing the Stroop task scan is necessary to explain the phenomenon.

There are a couple of technical issues that need to be discussed here. First of all, we conducted motion correction during preprocessing which has not been frequently reported in the literature in ICA analyses. We rationale that for the majority of functional connectivity analysis (e.g., seed-based connectivity analysis), in addition to the slice-time correction, motion correction, and normalization, filtering and regressing out covariates (such as six motion parameters, white matter signal, and CSF signal) are also essential during data preprocessing [40]. Regressing out estimated motion parameters and physiological signals can largely increase the gray matter temporal signal to noise [41]. We believe that it makes sense to include these preprocessing steps before ICA. We expect that performing ICA without these preprocessing steps would probably increase some independent components of noise. Secondly, one may concern how our interested components were influenced by the template which we used in the study. It is worth to note that the template provided by Laird and colleagues was based on a metadata set associated with 8637 functional brain imaging experiments across 31,724 subjects. We believe the key elements of each network do not deviate much from the template of other datasets [20], although the naming of the components is relatively different (e.g., the affective network that we named here was referred as “limbic” by Yeo and colleague). The number of ICs should have some influences on our results. How to describe/present neural networks and label their functions is one of the hot research areas and an ideal solution for mapping brain function may arrive in the very near future. In addition, the networks we are discussing in this study have covered several subnetworks. For example, our salience network included the bilateral insula and dorsal anterior cingulate. As indicated by Menon and Uddin [33], the anterior insula has different roles from the posterior insula. The anterior insula, not the posterior one, should be part of the salience network. Similarly, the function of subcomponents within DMN is also different [18], which could be the reason why we did not find a significant change in the DMN in depression patients compared with the controls. Therefore, instead of 20 components in the ICA analysis, using a larger number of components to define neural networks in a fine scale might leads to different conclusions.

In addition, we only studied the interactions between any two neural networks at a time. Using more complicated models that calculate the interactions among the networks simultaneously is necessary to confirm our results. We studied the internetwork correlations in geriatric depression because there have been known pathological disconnections in geriatric depression. Thus, our results cannot be generalized to younger depression patients. Because different regions might be involved in the pathology of geriatric depression due to large variations of outcomes from cerebrovascular diseases, further studies should be conducted to examine whether and how different cerebrovascular deficits affect our findings. This study is also limited by the small sample size and different medications of the depression patients. The small number of actively depressed patients may impact on the robustness of the significance of our results. This might explain why we only found significant alterations in the affective network but not in the executive network. Based on the results from the small number of patients, perhaps what we may conclude here is that at least deficits in the affective network were more robust and obvious than those in the executive network in the actively depressed group. Future replication studies in unmedicated patients with geriatric depression in a larger sample are warranted to confirm our conclusions.

While deficits of resting activity in depression have been reported in a number of studies in major depression, the aberrant interactions among intrinsic neural networks have not been demonstrated previously. Although our current study cannot determine which was the primary deficit in major depression firmly, the altered network activity, or the interactions among networks, we were able to examine the interactions between networks directly using the ICA approach. Our results have demonstrated that hyperactivity within the affective network (the automatic emotion regulation system), in particular the orbitofrontal cortex, in conjunction with sparse correlation among the central executive network, attentional network, and the salience network, is the core dysfunction of older depression patients during resting state. The results are in consistent with several depression models proposed in the literature and indicated that studying the correlations among networks is an effective approach in revealing neural mechanisms of depression.

## Supplementary Material

Figure S1 provides a complete view of each independent component (IC) shown in Figure 2. The Ics were generated from the data of all participants including both depression and never-depressed control individuals. Again, only the ICs that best matched to the components reported in the study of Laired and colleagues were reported in our study.

## Figures and Tables

**Table 1:** The clinical profiles of the participants.

	Depression (*n* = 32)	Control (*n* = 32)	*P* value
Gender (F/M)	18/14	19/13	0.80^+^
Age	68.3 (6.5)	71.8 (8.2)	0.06
Years of education	14.9 (3.1)	16.0 (2.5)	0.11
MADRS	7.0 (9.2)	0.0 (0.9)	<0.001^*^
Number medicated for hypotension	11	8	0.40^+^
Number medicated for antidepressants	18	0	
Monotherapy			
SSRI	4		
SARI	2		
SNRI	1		
Tricyclic	2		
Combined treatment			
Two SSRIs	4		
SSRI with either SARI or NDRI	2		
SARI & NDRI	2		
SNRI & NDRI	1		
Executive function (Stroop task)	−0.10 (0.82)	0.24 (0.71)	0.08

^+^Chi-square test, and the rests were two-sample *t*-tests; ^*^significant results with *P* < 0.05. SSRI = selective serotonin reuptake inhibitor; SARIs = serotonin antagonist and reuptake inhibitors; SNRIs = serotonin-norepinephrine reuptake inhibitors; NDRIs = norepinephrine-dopamine reuptake inhibitors.

**Table 2:** The clusters of each IC component identified matches the CEN, CAN, DMN, AN, and SN, respectively.

Network	IC	Clusters	Peak coordinate (MNI *X*, *Y*, *Z*)
DMN	IC1	Bilateral medial prefrontal cortex	[4, 59, −2]
		Bilateral posterior cingulate	[−7, −55, 19]; [1, 59, −5]
		Bilateral lateral parietal cortex	[−42, −65, 36]; [45, −63, 32]

AN	IC12	Bilateral dorsomedial prefrontal cortex	[−21, 35, 38]; [28, 42, 40]
		Rostral anterior cingulate	[6, 47, −4]
		Bilateral subgenual cingulate	[4, 41, −11]
	IC18	Bilateral subgenual cingulate	[13, 24, −18]
		Bilateral rostral anterior cingulate	[0, 61, 13]
		Bilateral ventrolateral prefrontal cortex	[−36, 37, 4]; [48, 32, 6]
		Bilateral orbitofrontal cortex	[−38, 34, −16]; [29, 36, −19]
		Bilateral amygdala	[−28, −1, −16]; [34, −3, −15]
		Bilateral caudate	[−6, 2, 8]; [10, 10, −2]

CEN	IC6	Left dorsolateral prefrontal cortex	[−46, 27, 19];
		Left dorsomedial prefrontal cortex	[−2, 23, 48]
		Bilateral superior parietal cortex	[−33, −50, 44]; [30, −58, 46]
		Right inferior temporal cortex	[−58, −43, −13]
		Left anterior part of posterior cingulate	[−5, −38, 38]
		Right cerebellum	[31, −69, −47]
	IC4	Right dorsolateral prefrontal cortex	[42, 22, 44]
		Right dorsomedial prefrontal cortex	[6, 27, 41]
		Bilateral superior parietal cortex	[−46, −50, 46]; [42, −58, 47]
		Right inferior temporal cortex	[61, −28, −6]
		Right anterior part of posterior cingulate	[5, −47, 40]
		Left cerebellum	[−39, −68, −45]

CAN	IC7	Bilateral frontal eye field	[−21, 2, 53]; [26, 2, 49]
		Bilateral precuneus	[−11, −69, 56]; [11, −65, 56]
		Bilateral parieto-occipital fissure	[−28, −82, 29]; [38, −73, 23]
		Bilateral lingual gyrus	[−13, −58, 11]; [18, −58, 15]

SN	IC10	Bilateral dorsal cingulate	[3, 27, 20];
		Bilateral insula	[−38, −14, 6]; [41, −12, 8]
		Bilateral parieto-occipital fissure	[−13, −62, 15]; [19, −57, 11]

**Table 3:** Mean (SD) inter-IC correlations that showed significant group differences and that correlated with performance of the Stroop task.

	Control	Depression	*P* value
IC12(AN)-IC6(CEN) correlation	0.30 (0.28)	0.15 (0.26)	0.03
IC12(AN)-IC7(CAN) correlation	−0.39 (0.33)	−0.22 (0.32)	0.04
IC12(AN)-IC10(SN) correlation	−0.18 (0.29)	−0.02 (0.32)	0.04
Stroop performance with IC10(SN)-IC6(CEN) correlation		*r* = −0.34	0.05
Stroop performance with IC10(SN)-IC18(AN) correlation		*r* = −0.51	0.003
