# Supplementary figures and images for: Altered Synchronizations among Neural Networks in Geriatric Depression
Source: Biomed Res Int. 2015 Jun 9;2015:343720. doi: 10.1155/2015/343720 (PMC4477114; doi:10.1155/2015/343720)

sFigure1

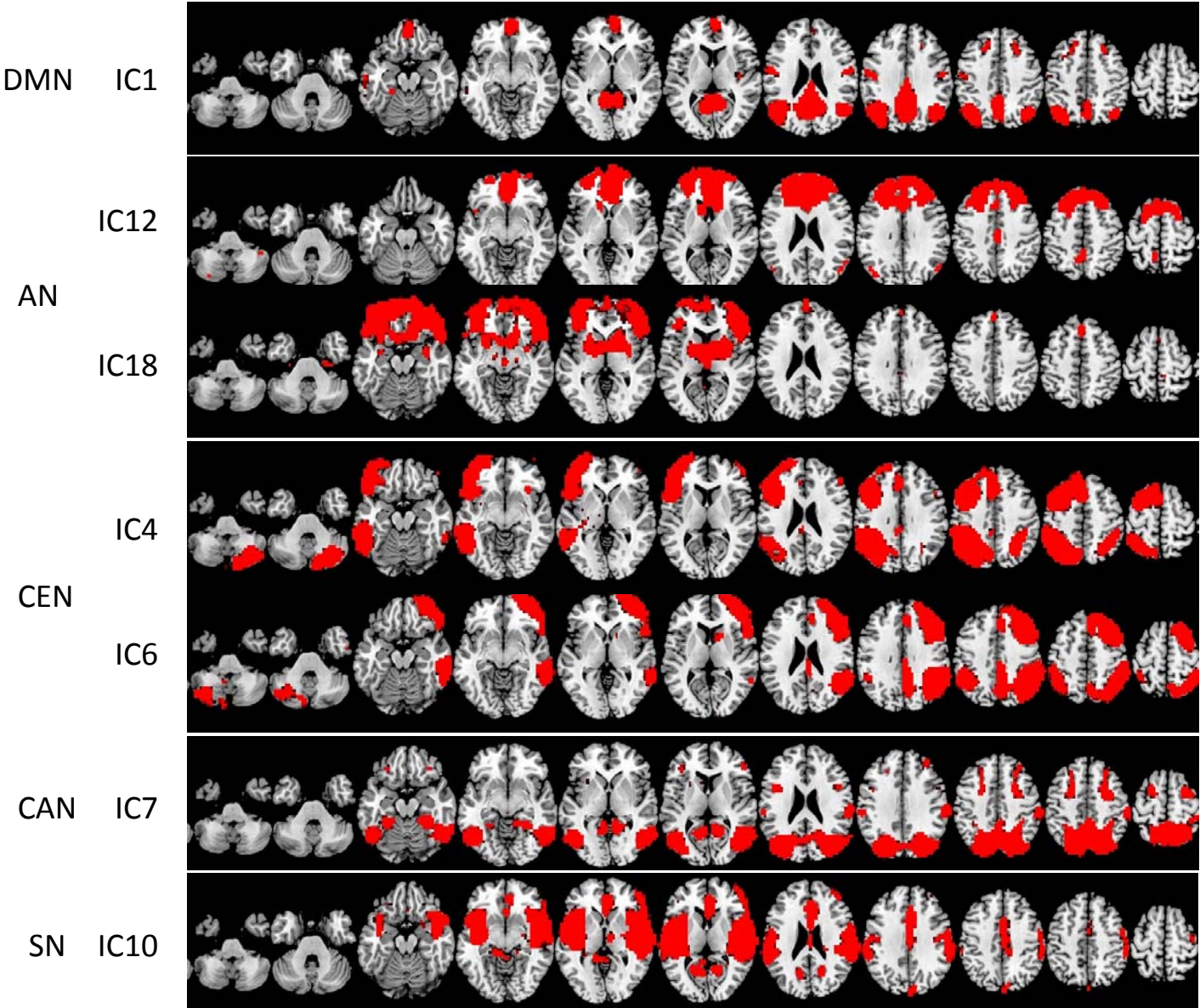

Supplement: Supplementary file 1 — Figure S1 provides a complete view of each independent component (IC) shown in Figure 2. The Ics were generated from the data of all participants including both depression and never-depressed control individuals. Again, only the ICs that best matched to the components reported in the study of Laired and colleagues were reported in our study. [file 343720.f1.pdf]
